# PP2 Ameliorates Renal Fibrosis by Regulating the NF-*κ*B/COX-2 and PPAR*γ*/UCP2 Pathway in Diabetic Mice

**DOI:** 10.1155/2021/7394344

**Published:** 2021-09-17

**Authors:** Jinying Wei, Xinna Deng, Yang Li, Runmei Li, Zhaohua Yang, Xiuyuan Li, Shan Song, Yonghong Shi, Huijun Duan, Haijiang Wu

**Affiliations:** ^1^Department of Pathology, Hebei Medical University, Shijiazhuang 050017, China; ^2^Departments of Oncology & Immunotherapy, Hebei General Hospital, Shijiazhuang 050051, China; ^3^Department of Immunology, Key Laboratory of Cancer Immunology and Biotherapy, National Clinical Research Center of Cancer, Tianjin Medical University Cancer Institute and Hospital, Tianjin 300060, China; ^4^Department of Foreign Language Teaching, Hebei Medical University, Shijiazhuang 050017, China; ^5^Medical Practice-Education Coordination & Medical Education Research Center, Hebei Medical University, Shijiazhuang 050017, China

## Abstract

Renal fibrosis is characterized by glomerulosclerosis and tubulointerstitial fibrosis in diabetic nephropathy (DN). We aimed to evaluate the effects of PP2 on renal fibrosis of DN. GSE33744 and GSE86300 were downloaded from the GEO database. Firstly, 839 DEGs were identified between nondiabetic and diabetic mice renal glomerular samples. COX-2 was selected to assess the effects of PP2 on renal glomerulosclerosis. In db/db mice, PP2 decreased the expression of COX-2, phosphorylated p65, and fibrotic proteins, accompanied with attenuated renal glomerulosclerosis. In cultured glomerular mesangial cells, high glucose- (HG-) induced p65 phosphorylation and COX-2 expression were attenuated by PP2 or NF-*κ*B inhibitor PDTC. PP2, PDTC, or COX-2 inhibitor NS-398 ameliorated abnormal proliferation and expression of fibrotic proteins induced by HG. Secondly, 238 DEGs were identified between nondiabetic and diabetic mice renal cortex samples. UCP2 was selected to assess the effects of PP2 on renal tubulointerstitial fibrosis. In db/db mice, PP2 decreased the expression of PPAR*γ* and UCP2, accompanied with attenuated renal tubulointerstitial fibrosis and EMT. In cultured proximal tubular cells, HG-induced PPAR*γ* and UCP2 expression was inhibited by PP2 or PPAR*γ* antagonist GW9662. PP2, GW9662, or UCP2 shRNA ameliorated HG-induced EMT. These results indicated that PP2 ameliorated renal fibrosis in diabetic mice.

## 1. Introduction

Diabetic nephropathy (DN), a major microvascular complication of diabetes mellitus, is the leading cause of end-stage renal disease. The pathogenesis and development of DN are extremely complicated. Clinically, it is initially characterized by an increase in urinary albumin excretion, followed by a gradual decline in glomerular filtration rate, eventually progressing to end-stage renal disease [1]. Morphologically, it is featured by thickening glomerular basement membrane and renal tubular basement membrane, widening mesangial matrix, glomerular sclerosis, podocyte loss, tubular atrophy and increased apoptosis, renal inflammatory infiltration, and renal interstitial fibrosis. In the meantime, periosteal capillaries become sparse, and the wall of efferent arteriole and afferent arteriole is hyaline degeneration, especially the efferent arteriole [2]. DN affects approximately one third of patients with either type I or type II diabetes, contributing to increased mortality and economic burden [3]. Therefore, it is extremely important to understand the molecular mechanisms of DN and identify novel therapeutic targets.

Renal fibrosis is the final common pathway for a variety of chronic kidney diseases, including DN. Chronic progressive renal fibrosis leads to loss of renal function and, eventually, end-stage renal disease. Generally, renal fibrosis is a complex process and refers to glomerulosclerosis and tubulointerstitial fibrosis, especially the increase of matrix protein synthesis and the inhibition of matrix degradation contributing to abnormal accumulation of extracellular matrix (ECM, e.g., collagen fibers and fibronectin) [4]. Many cells in the kidney have been reported to participate in this pathogenesis, especially renal mesangial cells and tubular epithelial cells. On the one hand, abnormal and excessive growth of mesangial cells plays a prominent role in the pathophysiologic processes of glomerulosclerosis in DN [5]. Under the diabetic conditions, mesangial cells are activated, leading to cell proliferation, glomerular hypertrophy, and excess renal ECM deposition in the glomerular mesangial region. Moreover, mesangial cells also secrete various inflammatory cytokines, adhesion molecules, chemokines, and enzymes, all of which contribute significantly to the process of renal glomerulosclerosis and reduced kidney function eventually [6]. On the other hand, tubular injury is another crucial determinant of progressive renal injury in DN, while epithelial-to-mesenchymal transition (EMT) of tubular cells contributes to tubulointerstitial fibrosis and glomerular tubular dissociation [7]. EMT, characterized by loss of epithelial phenotype and gain of profibrotic features that are characteristic of mesenchymal cells, enables cell cycle arrest of renal tubular epithelial cells, elevated migration capacity, resistance to apoptosis, the ability to produce inflammatory cytokines, and even direct production of ECM [8]. Nevertheless, the exact role of mesangial cells and tubular epithelial cells on the renal fibrosis and pathogenesis of DN has not been completely understood yet.

PP2 [4-amino-5-(4-chlorophenyl)-(t-butyl)pyrazolo[3,4-d]pyrimidine] is a selective Src family kinase inhibitor. c-Src is a member of the Src tyrosine kinase family that is involved in many cellular events such as mitosis, cell growth, and tumorigenesis [9, 10]. In response to a variety of external stimuli, c-Src can be activated (phosphorylated at Tyrosine 416) and subsequently mediates intracellular signal transduction by phosphorylating tyrosine residues of numerous cellular cytosolic, nuclear, and membrane proteins [2]. We and other investigators have verified the critical role of c-Src in pathological conditions including DN. In glomerular mesangial cells, astaxanthin treatment or overexpression of Cx43 attenuated the high glucose- (HG-) induced interaction between Nrf2 and c-Src, thereby enhancing the Nrf2 accumulation in the nuclei [11, 12]. In podocytes, c-Src mediated the translocation of diacylglycerol kinase *α* and contributed to the amelioration of DN upon stimulation by epigallocatechin gallate [13]. In addition, pretreatment of the differentiated podocytes with the c-Src kinase inhibitor, PP2, not only inhibited phosphorylation of c-Src but also restricted phosphorylation of EGFR and the activation of TGF*β*-Smad2/3 signaling pathway in response to HG [14]. In tubular epithelial cells, we have previously demonstrated that PP2 reduces c-Src activity and therefore inhibits cell apoptosis, which at least in part, is due to suppressed p38 MAPK activation [15]. However, the exact role of c-Src on renal fibrosis is yet to be elucidated.

In the present study, we aimed to investigate the role of c-Src on proliferation and fibrosis of mesangial cells as well as EMT of tubular epithelial cells in DN and identify its novel downstream genes. Therefore, we performed two separate studies to address these items. In the first study, by performing bioinformatics analysis based on Gene Expression Omnibus (GEO) data, we identified the differentially expressed genes (DEGs) between nondiabetic and diabetic renal glomerular samples. We identified COX-2 as the common hub gene of DM. Then, we performed a serial experiment to investigate the correlation between c-Src activation and COX-2 expression and evaluated the effects of c-Src/COX-2 pathway on proliferation and fibrosis of mesangial cells under in vivo and in vitro diabetic conditions. In the second study, by performing bioinformatic analysis based on GEO data, we identified the DEGs between nondiabetic and diabetic mice renal kidney cortex samples. We identified UCP2 as the common hub gene of DN. Then, we performed a serial experiment to investigate the correlation between c-Src activation and UCP2 expression and evaluated the effects of c-Src/UCP2 pathway on EMT of tubular epithelial cells under in vitro and in vivo diabetic conditions. The present study may provide an effective therapeutic target for DN.

## 2. Materials and Methods

### 2.1. Bioinformatic Analysis

Microarray gene expression profiles GSE33744 [16] and GSE86300 [17] were used to perform bioinformatic analysis, respectively. Probe IDs of two datasets were matched to gene symbol on the platform. Probe IDs matching to none or multiple genes symbol were deleted. If multiple IDs match a single gene, the highest expression value of these probes would be set for this gene. The limma package of *R* software program (version 4.0.3, https://www.r-project.org/) was used to normalize and screen for DEGs in each dataset, respectively. Adjusted *p* value <0.05 and ∣log FC | ≥1 were selected as the cut-off criteria of DEGs. The upregulated and downregulated DEGs were saved for subsequent analysis.

In order to explore the biological function and pathway of the above DEGs, Gene Ontology (GO) and Kyoto Encyclopedia of Genes and Genomes (KEGG) enrichment analysis were performed with the Database for Annotation, Visualization, and Integrated Discovery (DAVID v6.8, https://david.ncifcrf.gov/). The results of enrichment analyses were downloaded for subsequent analysis. *p* value <0.05 was considered statistically significant.

The STRING database (https://www.string-db.org/) was used to construct protein-protein interaction (PPI) network for the identified DEGs with the restriction of a minimum required interaction score of 0.400. The Cytoscape software (http://www.cytoscape.org/) was used to visualize the PPI network. The CytoHubba plugin in Cytoscape software was employed to identify the top 50 hub genes among DEGs. The Molecular Complex Detection (MCODE) plugin in Cytoscape software was employed to select the significant modules.

### 2.2. Materials

Antibodies against c-Src, phospho-Src (Tyr416), NF-*κ*B p65, phospho-NF-*κ*B p65 (Ser536), COX-2, PPAR*γ*, and UCP2 were purchased from Cell Signaling Technology (Beverly, MA). Antibodies against fibronectin and collagen IV were purchased from Santa Cruz Biotechnology (Santa Cruz, CA, USA). Antibodies against E-cadherin, *α*-SMA, DRP1, and FIS1 were purchased from the Proteintech Group (Chicago, USA). The *β*-actin antibody was purchased from Abcam (Cambridge, UK). The goat anti-rabbit IgG-HRP secondary antibody was purchased from Zhongshan Golden bridge Biotechnology (Beijing, China). c-Src inhibitor PP2, NF-*κ*B inhibitor PDTC, COX-2 inhibitor NS-398, and PPAR*γ* antagonist GW9662 were purchased from Selleck Chemicals (Houston, TX, USA). The pIRES2-ZsGreen1-UCP2 overexpression plasmid and PLV-UCP2-shRNA plasmid were purchased from Yingrun Biotechnology Inc. (Changsha, China). PhosSTOP phosphatase inhibitor cocktail tablets were purchased from Roche Ltd. (Mannheim, Germany). The protease inhibitor cocktail was purchased from Calbiochem (San Diego, CA, USA). All culture media were purchased from Gibco-BRL (Grand Island, NY, USA). D-glucose was purchased from Sigma (St. Louis, MO, USA). The polyvinylidene difluoride (PVDF) membrane was purchased from Millipore (Billerica, MA, USA).

### 2.3. Animals and Groups

The animal experiment was performed as previously described [15]. Briefly, male diabetic db/db mice and nondiabetic db/m mice were purchased from the Model Animal Research Center of Nanjing University. According to the guidelines of National Institute of Health, all experimental animals were housed in the specific pathogen-free conditions with free access to food and water. At eight weeks of age, the animals were randomly divided into three groups: control group (db/m mice, *n* = 10), diabetes group (db/db mice, *n* = 10), and PP2 group (db/db mice treated with PP2, *n* = 10). The PP2 group received 2 mg/kg PP2 dissolved in DMSO every other day by i.p. injection. Mice in the control group and diabetes group received the same amount of normal saline. At the age of 16 weeks, the animals that were sacrificed by exsanguination and the kidneys were harvested for further analysis.

### 2.4. Cell Culture

Immortalized mouse mesangial cells (MMCs) were obtained from the Chinese Academy of Sciences, Shanghai Institute for Biological Sciences Cell Resource Center and stored in our laboratory. MMCs were cultured in DMEM/F12 (3 : 1) medium supplemented with 10% fetal bovine serum, 1% streptomycin-penicillin mixture in a 95% air, and 5% CO_2_ atmosphere at 37°C. The culture medium was replaced every two days. After reaching 70-80% confluence, the cells were washed once with serum-free DMEM medium and then growth arrested in serum-free DMEM medium for 24 h to synchronize the cell growth. The media was then changed to fresh serum-free media containing normal glucose (NG, 5.6 mmol/l) or HG (30 mmol/l) for an additional 48 h. PP2 (10 *μ*M), PDTC (10 *μ*M), or NS-398 (20 *μ*M) was added to the culture medium 2 h prior to HG.

Human renal tubule epithelial (HK-2) cells were obtained from American Type Culture Collection (ATCC, Manassas, VA, USA) and cultured in DMEM medium supplemented with 10% fetal bovine serum, 1% streptomycin-penicillin mixture in a 95% air and 5% CO_2_ atmosphere at 37°C. The culture medium was replaced every two days. After reaching 70-80% confluence, the cells were washed once with serum-free DMEM medium, and then growth-arrested in serum-free DMEM medium for 24 h to synchronize the cell growth. The media was then changed to fresh serum-free media containing NG (5.6 mmol/l) or HG (30 mmol/l) for an additional 48 h. PP2 (10 *μ*M) and GW9662 (20 *μ*M) were added to the culture medium 2 h prior to HG. Stable transfections of HK-2 cells with pIRES2-ZsGreen1-UCP2 plasmid or PLV-UCP2-shRNA plasmid were performed with Lipofectamine 2000 (Invitrogen, Carlsbad, CA) according to the manufacturer's instructions.

### 2.5. Protein Extraction and Western Blotting

Whole protein extraction was performed according to the method used in the previous research [15]. In detail, after the treatment, renal cortex tissues and cells were washed twice with ice cold phosphate-buffered saline (PBS), lysed in protein lysis buffer with protease and phosphatase inhibitors for 30 min on ice. The cell lysates were centrifuged at 12,000 rpm for 20 min at 4°C. Protein concentration was determined with the BCA protein assay kit with BSA as the standard. Protein samples (40 mg/lane) were separated with SDS-PAGE and transferred to PVDF membranes which were then blocked with 5% (v/v) nonfat milk at 37°C for 2 h. The membranes were subsequently incubated overnight at 4°C with primary antibodies against c-Src, phospho-Src (Tyr416), p65, phospho-p65 (Ser536), COX-2, PPAR*γ*, UCP2, fibronectin, collagen IV, E-cadherin, *α*-SMA, DRP1, FIS1, and *β*-actin, respectively. The next morning, the membranes were rinsed with TBST and incubated with secondary antibody for 1 h at room temperature. Imaging was performed with the Odyssey Fc System (LICOR, USA). The intensity of the bands was performed with NIH ImageJ software.

### 2.6. Histology and Immunohistochemistry

Kidneys were fixed in 4% paraformaldehyde overnight and embedded in paraffin. Paraffin sections were cut at 2 mm and processed for periodic acid Schiff (PAS) and Masson's trichrome staining according to the standard protocol. For immunohistochemical analysis, paraffin sections were cut at 4 mm and deparaffinized with xylene and rehydrated in graded ethanol. Internal peroxidase was inactivated with 3% hydrogen peroxide in 100% methanol for 30 min. Antigen retrieval was performed with microwave oven in 10 mM citrate buffer for 15 min. 10% normal goat serum in PBS was then added to the sections for 30 min at room temperature to block nonspecific antibody binding. The sections were then incubated with primary antibodies for fibronectin, collagen IV, E-cadherin, *α*-SMA, and COX-2 (diluted 1 : 300) overnight at 4°C. After rinsed in PBS, the sections were then incubated with biotinylated secondary antibody and horseradish peroxidase-conjugated streptavidin. Labeling was visualized with diaminobenzidine to produce a brown color, and sections were counterstained with hematoxylin. For negative controls, the primary antibody was omitted. The area percent of staining was calculated using ImageJ software.

### 2.7. Transmission Electron Microscopy

The ultramicrostructure of glomerulus was observed with electron microscopy. Kidney tissue was immerged in 2.5% (v/v) glutaraldehyde and 1% (v/v) osmium tetroxide. Then, the kidney tissue was dehydrated in graded alcohol solutions and embedded in Epon 812 resin. Ultrathin sections (70-80 nm) were cut and double-stained with uranyl acetate and lead citrate. Sections in ultrastructure were observed and photographed using Hitachi H7500 transmission electron microscope (Hitachi, Tokyo, Japan).

### 2.8. Cell Proliferation Assay

Cell proliferation of MMCs was estimated with the 3-(4,5-dimethylthiazol-2-yl)-2,5-diphenyl tetrazolium (MTT) assay according to the manufacturer's instructions (Beyotime Biotechnology, Shanghai, China). In brief, MMCs (2000 cells/well) were inoculated into 96-well plates. After adherence, the medium was replaced with serum-free medium for 24 h. The media was then changed to fresh serum-free media containing NG (5.6 mmol/l) or HG (30 mmol/l) for an additional 24 h. PP2 (10 *μ*M), PDTC (10 *μ*M), or NS-398 (20 *μ*M) was added to the culture medium 2 h prior to HG. Next, 10 *μ*l MTT solution (5 mg/ml) was added to each well and incubated for 4 h at 37°C. Then, 100 *μ*l formazan solution was added to each well and incubated for 4 h at 37°C to fully dissolve the crystals. Finally, the absorbance was measured at 570 nm using BioTek's Microplate spectrophotometer (Gene Company Limited, USA). All conditions were carried out in triplicate (*n* = 6), and the experiments were replicated three times.

### 2.9. Mitochondrial Membrane Potential (MMP) Detection

MMP was detected with the MMP Assay Kit with JC-1 (Solarbio, Beijing, China). After cultured in 6-well plates under the different experimental conditions for 48 h, HK-2 cells were washed in PBS and incubated with JC-1 dye for 20 min at 37°C. After the dye was removed by washing, the cells were immediately imaged using a TCS SP8 confocal microscope (Leica Microsystems, Wetzlar, Germany). JC-1 monomers emit a green fluorescence, while aggregates of JC-1 show intense red fluorescence. In apoptotic cells, JC-1 does not form aggregates in mitochondria with low MMP and exhibit green fluorescence.

### 2.10. Mitochondrial Morphology Analysis

After cultured in 6-well plates under the different experimental conditions for 48 h, HK-2 cells were washed in PBS and incubated with MitoTracker Red CMXRos (Beyotime Biotechnology, Shanghai, China) using a working concentration of 200 nM at 37°C for 30 min. After the dye was removed by washing, the cells were immediately imaged using a TCS SP8 confocal microscope (Leica Microsystems, Wetzlar, Germany).

### 2.11. Statistical Analysis

Data are expressed as means ± SD. Statistical analysis was performed by one-way ANOVA. Statistical significance was defined as *p* < 0.05.

## 3. Results

### 3.1. Identification of COX-2 as a DEG between Nondiabetic and Diabetic Mice Renal Glomerular

Microarray gene expression profile GSE33744 was downloaded from the GEO database (https://www.ncbi.nlm.nih.gov/geo/). The platform for GSE33744 is GPL1261 [Mouse430_2] Affymetrix Mouse Genome 430 2.0 Array. This dataset contains 39 samples, and we selected 5 glomeruli samples from nondiabetic BKS db/m mouse and 5 glomeruli samples from diabetic BKS db/db mouse to perform bioinformatic analysis.

**Figure 1:**
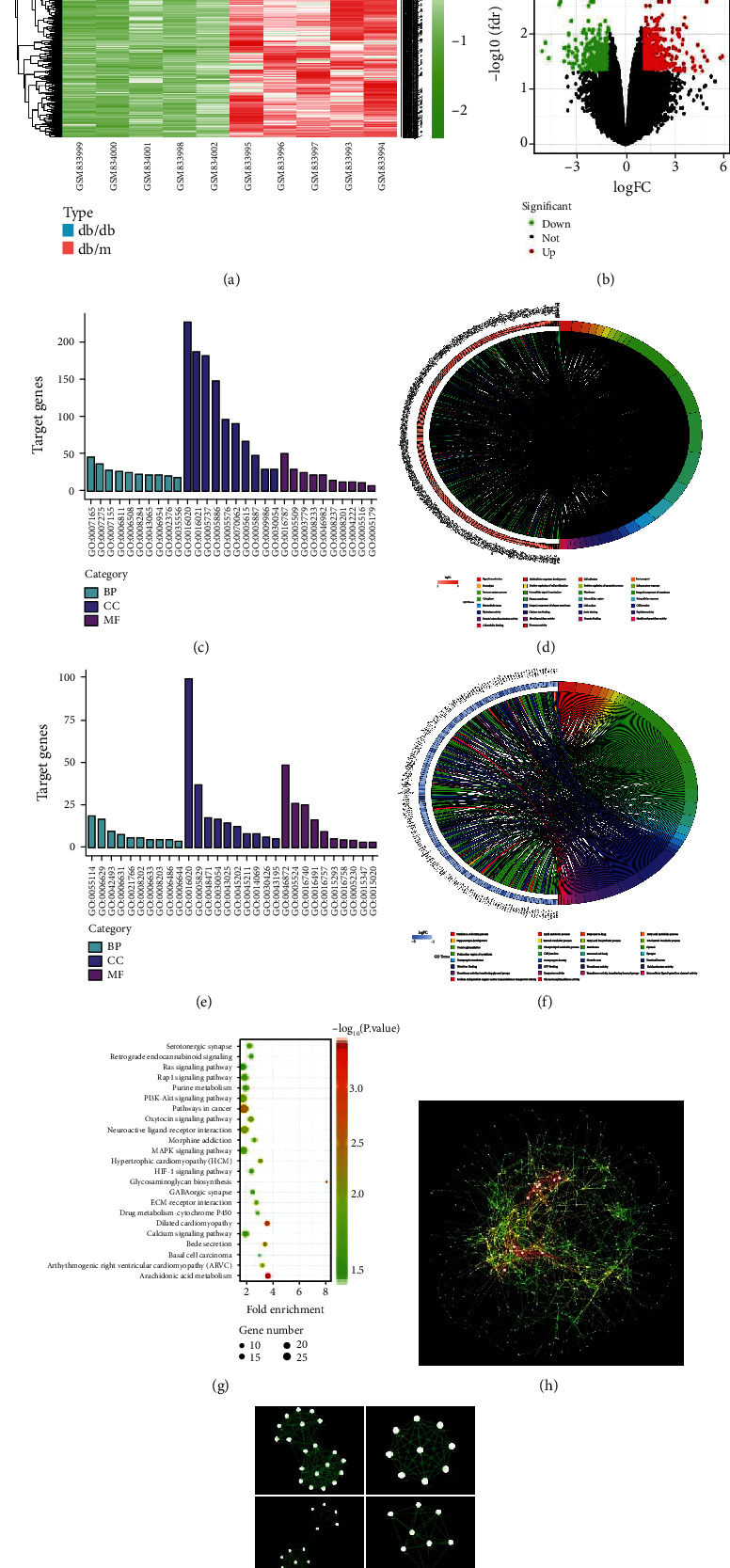
Identification of DEGs and biological pathways between nondiabetic and diabetic mice renal glomerular in GSE33744. (a) Heatmap of DEGs. Red represents an upregulated gene, and green represents a downregulated gene. Legend on the top right indicates the log fold change of the genes. (b) Volcano plot of DEGs. Red represents an upregulated gene, and green represents a downregulated gene. Black represents no significant change in gene expression. (c) Top 30 enriched GO terms of upregulated DEGs. (d) Distribution of upregulated DEGs in different GO-enriched functions. (e) Top 30 enriched GO terms of downregulated DEGs. (f) Distribution of downregulated DEGs for different GO-enriched functions. (g) KEGG pathway enrichment analysis of DEGs. (h) PPI network of DEGs. Every node represents a gene-encoded protein, and each edge represents the interaction between them. The edge color represents the core degree of protein. (i) Top 6 functional modules identified from the PPI network.

Based on the criteria of adjusted *p* value <0.05 and ∣log FC | ≥1, a total of 839 DEGs were identified from GSE33744. Among them, 579 were upregulated, and 260 were downregulated in diabetic mice renal glomerular. The DEGs are visualized by the cluster heat map (Figure 1(a)) and the volcano plot (Figure 1(b)). GO functional enrichment analysis of DEGs was categorized based on biological processes, cellular components, and molecular functions. The top 10 enriched GO terms of biological processes, cellular components, and molecular functions for the upregulated and downregulated DEGs were displayed in Figures 1(c)–1(f). The detailed information on the GO analysis of the upregulated and downregulated DEGs are list in Table 1. In the upregulated DEGs, the most enriched GO terms of biological processes, cellular components, and molecular functions were associated with signal transduction, membrane, and hydrolase activity, respectively. In the downregulated DEGs, the most enriched GO terms of biological processes, cellular components, and molecular functions were associated with oxidation-reduction process, membrane, and metal ion binding, respectively. KEGG pathway enrichment analysis demonstrated that the DEGs were enriched in 23 pathways (Figure 1(g)). The detailed information on the KEGG enrichment analysis of DEGs are listed in Table 2.

PPI network of DEGs was constructed with Cytoscape software, and the results are shown in Figure 1(h). Furthermore, 23 functional modules were identified from the PPI network with MCODE plugin of Cytoscape, and the 6 most important modules are shown in Figure 1(i). Afterward, the top 50 hub genes were identified from the PPI network with the CytoHubba plugin of Cytoscape based on their degree value, and the results are shown in Table 3. The top 2 hub genes were epidermal growth factor receptor (EGFR) and COX-2 (also known as prostaglandin-endoperoxide synthase 2, PTGS2). The role of the c-Src/EGFR signaling pathway in DN has been previously reported [18]. COX-2 is an inducible enzyme responsible for prostaglandin synthesis and primarily associated with inflammation. There is also increasing evidence that COX-2 is upregulated in pathological conditions [19]. The GO pathway enrichment showed that COX-2 was related to positive regulation of apoptotic process, inflammatory response, positive regulation of cell proliferation, response to lipopolysaccharide, oxidation-reduction process, response to oxidative stress, angiogenesis, negative regulation of cell proliferation, regulation of blood pressure, cellular response to mechanical stimulus, memory, regulation of cell proliferation, maintenance of blood-brain barrier, prostaglandin biosynthetic process, positive regulation of NF-kappaB import into nucleus, positive regulation of synaptic transmission, glutamatergic, positive regulation of vascular endothelial growth factor production, positive regulation of smooth muscle contraction, positive regulation of nitric oxide biosynthetic process, positive regulation of vasoconstriction, positive regulation of cell death, positive regulation of phosphatidylinositol 3-kinase signaling, sensory perception of pain, positive regulation of smooth muscle cell proliferation, cyclooxygenase pathway, positive regulation of fever generation, response to lithium ion, negative regulation of calcium ion transport, prostaglandin metabolic process, membrane, endoplasmic reticulum, intracellular membrane-bounded organelle, neuron projection, organelle membrane, metal ion binding, oxidoreductase activity, heme binding, and prostaglandin-endoperoxide synthase activity. Therefore, COX-2 pathway was selected for further investigation.

### 3.2. Inhibition of c-Src Ameliorates ECM Accumulation and Fibrosis via NF-*κ*B/COX-2 Pathway in Renal Glomerulus of Diabetic Mice

**Figure 2:**
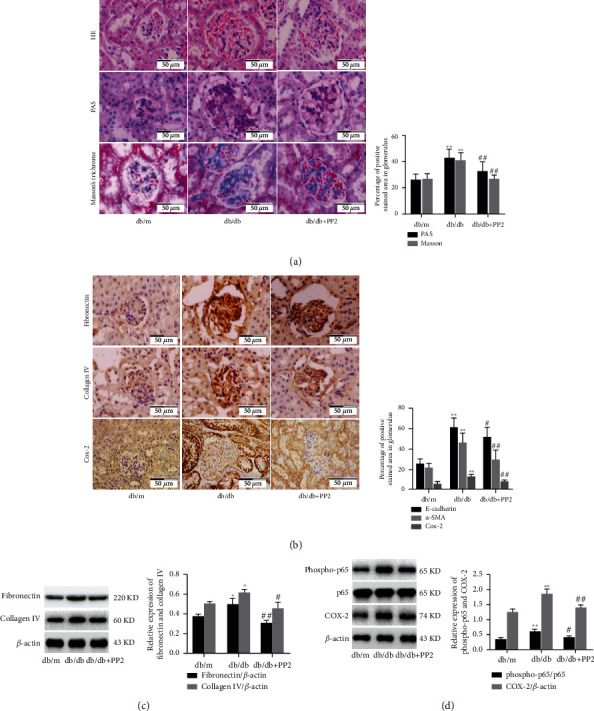
Effect of c-Src inhibiton on ECM accumulation and fibrosis in the renal glomerulus of diabetic mice. (a) HE, PAS, and Masson's trichrome staining of kidney sections (original magnification ×400). (b) Immunohistochemical staining of kidney sections with fibronectin and collagen IV (original magnification ×400). (c) The expression levels of fibronectin and collagen IV analyzed by western blotting. (d) The expression levels of phospho-p65, p65, and COX-2 analyzed by western blotting. The number of mice in every group was 10. 10 visual fields were randomly selected from each group, and the area percent of staining was calculated for statistical analysis. All western blotting experiments were performed independently at least in triplicate. Values are expressed as means ± SD. ^∗^*p* < 0.05 versus db/m group. ^∗∗^*p* < 0.01 versus db/m group. ^#^*p* < 0.05 versus db/db group. ^##^*p* < 0.01 versus db/db group.

Our previous study has shown that PP2 treatment can attenuate diabetes induced c-Src activation and therefore ameliorates renal tubular epithelial cells apoptosis in db/db mice [15]. To understand whether c-Src inhibition has a protective effect in renal fibrosis, we first explored the role of PP2 treatment on the histopathological changes of kidney. As shown in Figure 2(a), HE staining showed that compared with the nondiabetic mice, glomerular hypertrophy, partial glomerulosclerosis, mesangial cells hyperplasia and hypertrophy, and inflammatory cell infiltration were significantly increased in diabetic mice. These pathological changes were significantly attenuated by PP2 treatment. Meanwhile, the results of PAS staining showed that there was prominent mesangial expansion in diabetic mice compared with the nondiabetic mice, which was ameliorated with PP2 treatment. Masson's trichrome staining and quantitative assessment confirmed that PP2 treatment resulted in a significant reduction in collagen deposition of renal glomerular when compared with the diabetic mice. Likewise, immunohistochemistry staining showed that ECM components, including fibronectin and collagen IV, were markedly increased in db/db mice than that in the db/m mice, whereas PP2 treatment significantly reversed these changes (Figure 2(b)). Western blotting results were consistent with the immunofluorescence observation (Figure 2(c)). These results indicate that c-Src plays an important role in the renal fibrosis and injury of diabetic mice.

As mentioned above, the results of the bioinformatic analysis indicated that COX-2 showed significantly different expression between nondiabetic and diabetic mice renal glomerular,and was implicated in the biological processes of positive regulation of NF-*κ*B import into nucleus. Then, we evaluated the relationship between c-Src inhibition and NF-*κ*B/COX-2 pathway in db/db mice. Western blotting showed that the phosphorylation levels of NF-*κ*B p65 (activation) and expression of COX-2 were markedly increased in db/db mice than that in the db/m mice, whereas PP2 treatment significantly reversed these changes (Figure 2(d)). Likewise, immunohistochemistry staining showed that the COX-2 expression was statistically increased in renal glomeruli of db/db mice than in the db/m mice, whereas PP2 treatment statistically reversed these changes (Figure 2(e)). Collectively, these data indicate that c-Src inhibition can attenuate renal ECM accumulation and fibrosis through regulation of NF-*κ*B activation and COX-2 expression in diabetic mice.

### 3.3. Inhibition of c-Src Ameliorates HG-Induced ECM Accumulation via NF-*κ*B/COX-2 Pathway in MMCs

**Figure 3:**
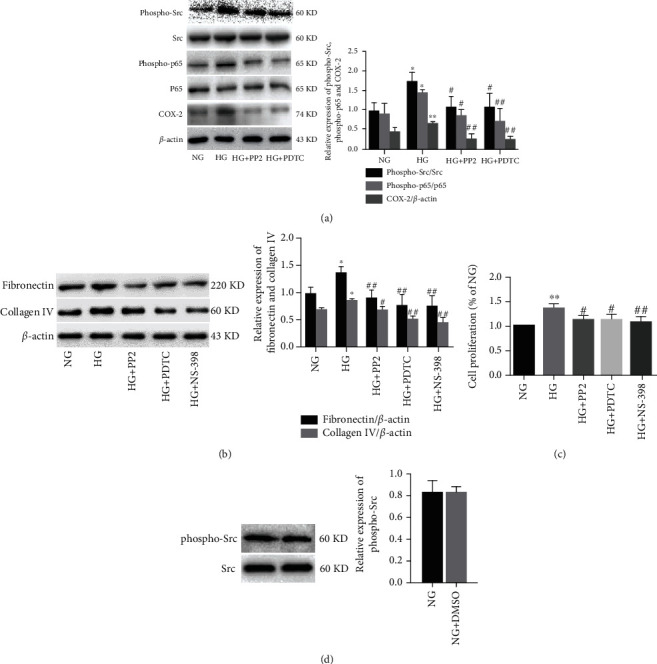
Effect of c-Src inhibiton on HG-induced ECM accumulation in MMCs. (a) The expression levels of phospho-Src, Src, phospho-p65, p65, and COX-2 analyzed by western blotting. (b) The expression levels of fibronectin and collagen IV analyzed by western blotting. (c) Cell proliferation of MMCs detected by the MTT assay. (d) The expression levels of phospho-Src and Src analyzed by western blotting. All experiments were performed independently at least in triplicate. Values are expressed as means ± SD. ^∗^*p* < 0.05 versus NG group. ^∗∗^*p* < 0.01 versus NG group. ^#^*p* < 0.05 versus HG group.^##^*p* < 0.01 versus HG group.

To determine whether the activation of c-Src is indispensable for HG-induced NF-*κ*B activity and COX-2 expression, we treated MMCs with the c-Src inhibitor PP2 or NF-*κ*B inhibitor PDTC. As shown in Figure 3(a), compared with NG groups, exposure of MMCs to HG for 48 h significantly increased phosphorylation of c-Src at Tyr416 and NF-*κ*B p65 at Ser536, which were significantly inhibited by PP2 or PDTC, respectively. PP2 and PDTC also markedly reduced the HG-induced COX-2 expression. We further assessed whether the c-Src/NF-*κ*B/COX-2 pathway was involved in HG-induced ECM accumulation in MMCs. As shown in Figure 3(b), compared with NG groups, exposure of MMCs to HG for 48 h significantly increased the expression of fibronectin and collagen IV, which could be significantly attenuated by PP2, PDTC, and NS-398, respectively. We further assessed whether the c-Src/NF-*κ*B/COX-2 pathway was involved in HG-induced MMCs proliferation with the MTT assay. As shown in Figure 3(c), exposure of MMCs to HG increased the cells' proliferation. The effects of HG on MMC proliferation were abolished by PP2, PDTC, and NS-398 treatment, respectively. Collectively, inhibition of c-Src/NF-*κ*B/COX-2 pathway significantly inhibited HG-induced ECM accumulation, inflammation, and proliferation in mesangial cells. In addition, as a solvent control for PP2, DMSO had no effect on activation of c-Src (Figure 3(d)).

### 3.4. Identification of UCP2 as a DEG between Nondiabetic and Diabetic Mice Renal Cortex

Microarray gene expression profile GSE86300 was downloaded from the GEO database (https://www.ncbi.nlm.nih.gov/geo/). The platform for GSE86300 is GPL7546 Affymetrix GeneChip Mouse Genome 430 2.0 Array [CDF: Mm_ENTREZG_10]. This dataset contains 10 samples, and 5 renal cortex samples from nondiabetic BKS db/m mouse and 5 renal cortex samples from diabetic BKS db/db mouse were used to perform bioinformatic analysis.

**Figure 4:**
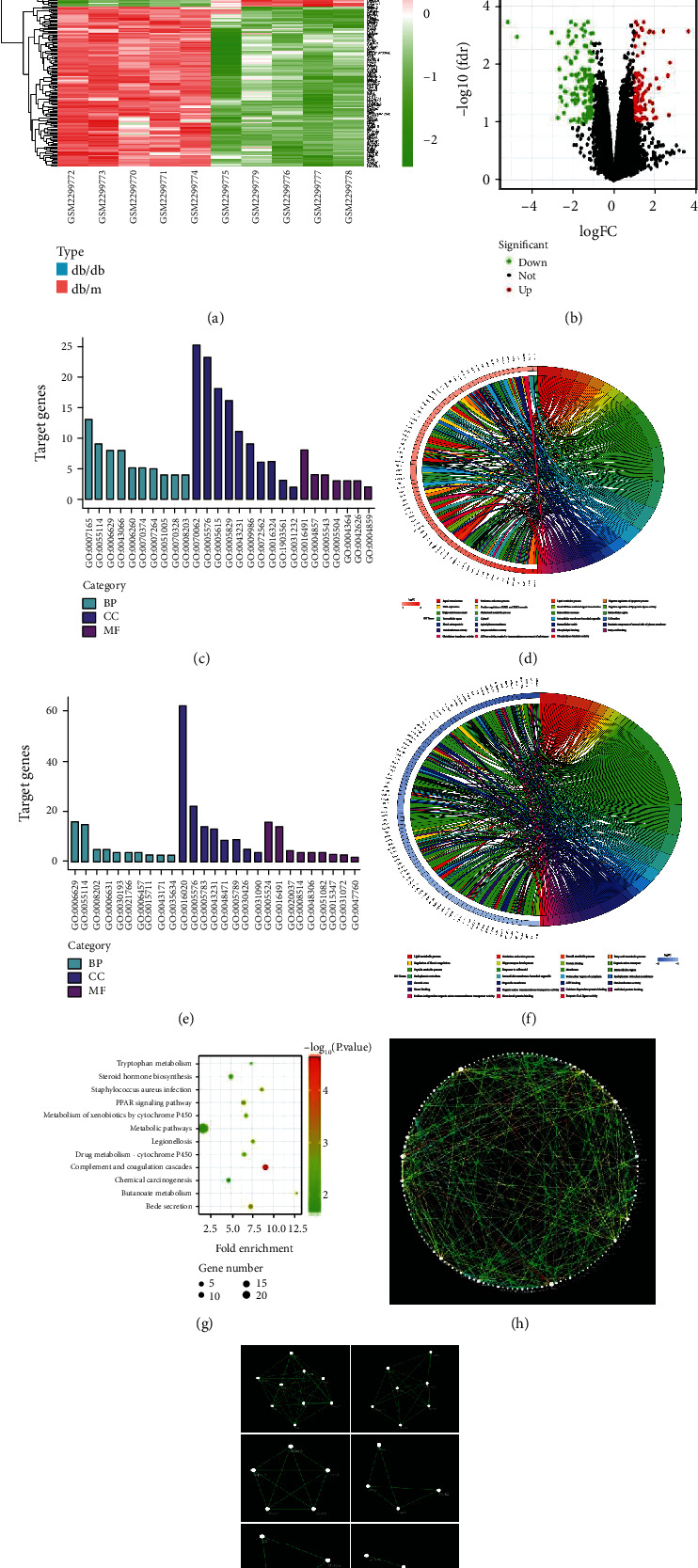
Identification of DEGs and biological pathways between nondiabetic and diabetic mice renal glomerular in GSE86300. (a) Heatmap of DEGs. Red represents an upregulated gene, and green represents a downregulated gene. Legend on the top right indicates the log fold change of the genes. (b) Volcano plot of DEGs. Red represents an upregulated gene, and green represents a downregulated gene. Black represents no significant change in the gene expression. (c) Top 30 enriched GO terms of upregulated DEGs. (d) Distribution of upregulated DEGs in different GO-enriched functions. (e) Top 30 enriched GO terms of downregulated DEGs. (f) Distribution of downregulated DEGs for different GO-enriched functions. (g) KEGG pathway enrichment analysis of DEGs. (h) PPI network of DEGs. Every node represents a gene-encoded protein, and each edge represents the interaction between them. The edge color represents the core degree of protein. (i) Top 6 functional modules identified from the PPI network.

Based on the criteria of adjusted *p* value <0.05 and ∣log FC | ≥1, a total of 238 DEGs were identified from GSE33744. Among them, 112 were upregulated, and 126 were downregulated in diabetic mice renal cortex. The DEGs are visualized by the cluster heat map (Figure 4(a)) and the volcano plot (Figure 4(b)). GO functional enrichment analysis of DEGs was categorized based on biological processes, cellular components, and molecular functions. The top 10 enriched GO terms of biological processes, cellular components, and molecular functions for the upregulated and downregulated DEGs were displayed in Figures 4(c)–4(f). The detailed information on the GO analysis of the upregulated and downregulated DEGs are list in Table 4. In the upregulated DEGs, the most enriched GO terms of biological processes, cellular components, and molecular functions were associated with signal transduction, extracellular exosome, and oxidoreductase activity, respectively. In the downregulated DEGs, the most enriched GO terms of biological processes, cellular components, and molecular functions were associated with lipid metabolic process, membrane, and ATP binding, respectively. KEGG pathway enrichment analysis demonstrated that the DEGs were enriched in 12 pathways (Figure 4(g)). The detailed information on the KEGG enrichment analysis of DEGs are listed in Table 5.

PPI network of DEGs was constructed using Cytoscape software, and the results are shown in Figure 4(h). Furthermore, 7 functional modules were identified from the PPI network using MCODE plugin of Cytoscape, and the 6 most important modules are shown in Figure 4(i). Afterward, the top 50 hub genes were identified from the PPI network with the CytoHubba plugin of Cytoscape based on their degree value, and the results are shown in Table 6. In these hub genes, UCP2 was annotated as an inner mitochondrial membrane protein that belongs to the uncoupling protein family and has recently been demonstrated in the control of mitochondria dysfunction [20]. Considering the key role of mitochondrial dysfunction in DN, we focused our attention to UCP2. The GO pathway enrichment showed that UCP2 was related to the biological processes of negative regulation of apoptotic process, liver regeneration. Therefore, the UCP2 pathway was selected for further investigation.

### 3.5. Inhibition of c-Src Ameliorates EMT and Tubulointerstitial Fibrosis via PPAR*γ*/UCP2 Pathway in Renal Tubule of Diabetic Mice

**Figure 5:**
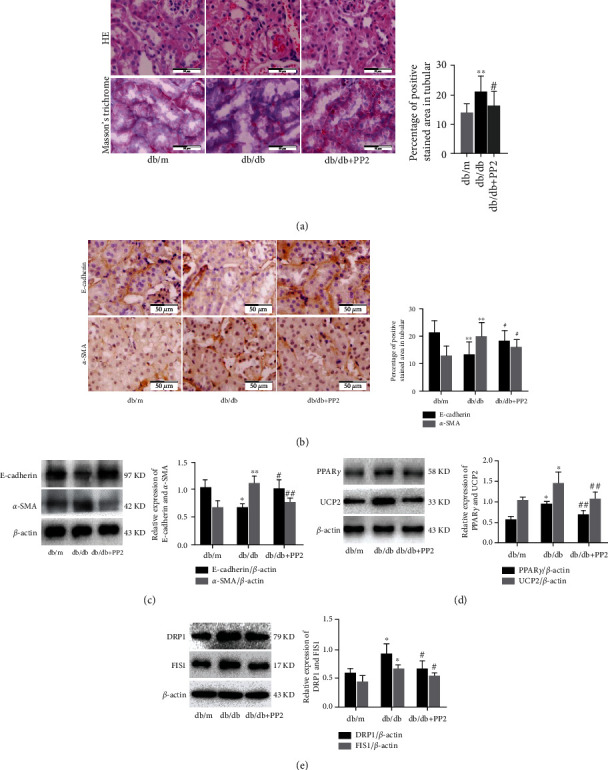
Effect of c-Src inhibiton on EMT and tubulointerstitial fibrosis in renal tubule of diabetic mice. (a) HE and Masson's trichrome staining of kidney sections (original magnification ×400). (b) Immunohistochemical staining of kidney sections with E-cadherin and *α*-SMA (original magnification ×400). (c) The expression levels of E-cadherin and *α*-SMA analyzed by western blotting. (d) The expression levels of PPAR*γ* and UCP2 analyzed by western blotting. (e) The expression levels of DRP1 and FIS1 analyzed by western blotting. The number of mice in every group was 10. 10 visual fields were randomly selected from each group, and the area percent of staining was calculated for statistical analysis. All western blotting experiments were performed independently at least in triplicate. Values are expressed as means ± SD. ^∗^*p* < 0.05 versus db/m group. ^∗∗^*p* < 0.01 versus db/m group. ^#^*p* < 0.05 versus db/db group. ^##^*p* < 0.01 versus db/db group.

Our previous studies have indicated that EMT in renal tubular epithelial cells, characterized by loss of epithelial phenotype and gain of profibrotic features that are characteristic of mesenchymal cells, is directly involved in the progression of tubulointerstitial fibrosis that eventually contributed to renal fibrosis in DN [7, 21]. To understand whether c-Src inhibition has a protective effect in renal EMT and tubulointerstitial fibrosis, we first explore the role of PP2 treatment on the histopathological changes of kidney. As shown in Figure 5(a), HE staining showed that compared with the nondiabetic mice, interstitial fibrous tissue proliferation and inflammatory cell infiltration, tubular ectasia, and cell swelling were significantly increased in diabetic mice. These pathological changes were significantly attenuated by PP2 treatment. Masson's trichrome staining and quantitative assessment confirmed that PP2 treatment resulted in a significant reduction in interstitial fibrosis when compared with the diabetic mice. Additionally, compared with the nondiabetic mice, transmission electron microscopy showed nuclear volume reduction (pyknosis), chromatin condensation, and nuclear membrane shrinkage in renal tubular epithelial cell of db/db mice. PP2 treatment could partially reverse these morphological changes of apoptosis in renal tubular epithelial cells. Likewise, immunohistochemistry staining showed that the expression of epithelial marker E-cadherin was markedly decreased, and mesenchymal marker *α*-SMA was markedly increased in db/db mice than that in the db/m mice, whereas PP2 treatment significantly reversed these changes (Figure 5(b)). Western blotting results were consistent with the immunofluorescence observation (Figure 5(c)). These results indicate that c-Src plays an important role in the renal EMT and tubulointerstitial fibrosis of diabetic mice.

As mentioned above, the results of the bioinformatic analysis indicated that UCP2 showed significantly different expression between nondiabetic and diabetic mice renal cortex. Interestingly, PPAR*γ* was previously highlighted as the major regulator of UCP2 under physiological conditions [22, 23]. Therefore, we evaluated the relationship between c-Src inhibition and PPAR*γ*/UCP2 pathway in db/db mice. Western blotting showed that the expression of PPAR*γ* and UCP2 was markedly increased in db/db mice than that in the db/m mice, whereas PP2 treatment significantly reversed these changes (Figure 5(d)). Moreover, western blotting also showed that the expression of DRP1 and FIS1, two mitochondrial fission-related proteins, was markedly increased in db/db mice than that in the db/m mice, whereas PP2 treatment significantly reversed these changes (Figure 5(e)). Collectively, these data indicate that c-Src inhibition can attenuate renal EMT and tubulointerstitial fibrosis through regulation of PPAR*γ* and UCP2 expression in diabetic mice.

### 3.6. Inhibition of c-Src Ameliorates HG-Induced EMT via PPAR*γ*/UCP2 Pathway in HK-2 Cells

**Figure 6:**
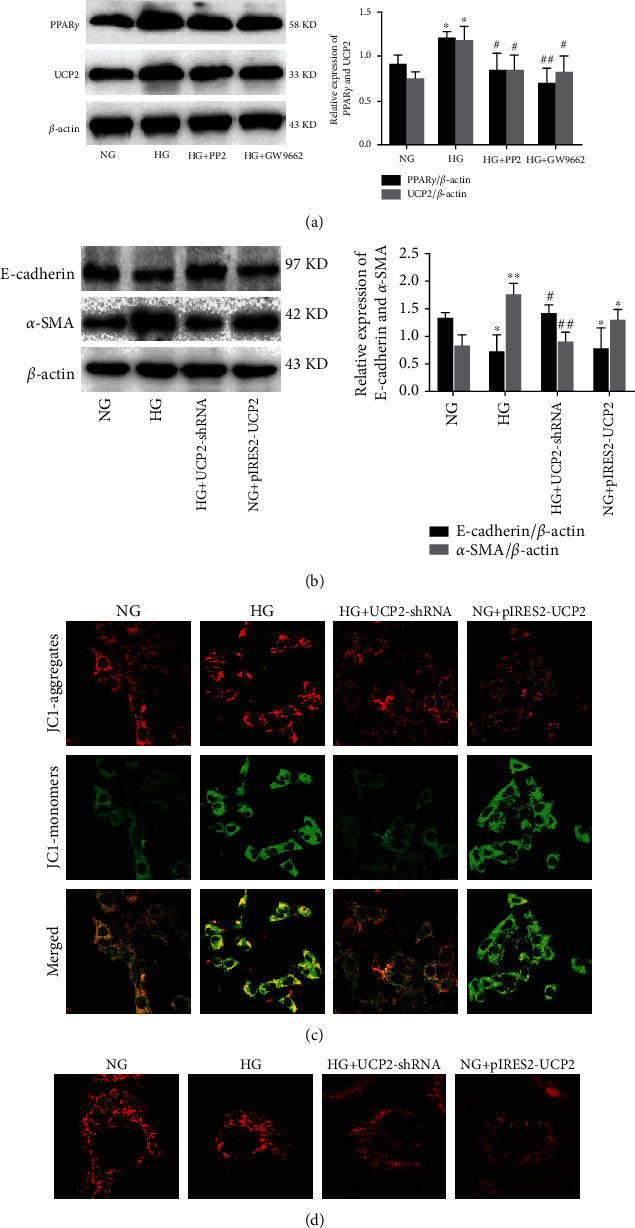
Effect of c-Src inhibiton HG-induced EMT in HK-2 cells. (a) The expression levels of PPAR*γ* and UCP2 analyzed by western blotting. (b) The expression levels of E-cadherin and *α*-SMA analyzed by western blotting. (c) MMP detected by the JC-1 membrane potential assay (magnification ×100). The shift of fluorescence from red to green after HG exposure indicates a decrease in mitochondria membrane potential. (d) The mitochondrial morphology detected by Mitotracker (magnification×400). All western blot experiments were performed independently at least in triplicate. Values are expressed as means ± SD. ^∗^*p* < 0.05 versus NG group. ^∗∗^*p* < 0.01 versus NG group. ^#^*p* < 0.05 versus HG group.^##^*p* < 0.01 versus HG group.

To determine whether the activation of c-Src is indispensable for HG-induced PPAR*γ* and UCP2 expression, we treated HK-2 cells with the c-Src inhibitor PP2 or PPAR*γ* antagonist GW9662. Compared with NG groups, exposure of HK-2 cells to HG for 48 h significantly increased PPAR*γ* and UCP2 expression, which were significantly inhibited by PP2 or GW9662, respectively (Figure 6(a)). We further assessed whether the c-Src/PPAR*γ*/UCP2 pathway was involved in HG-induced EMT in HK-2 cells. As shown in Figure 6(b), compared with NG groups, exposure of HK-2 cells to HG or UCP2 overexpression plasmid for 48 h significantly decreased the expression of E-cadherin and increased the expression of *α*-SMA, respectively. Furthermore, HG-induced EMT could be significantly attenuated by transfection with UCP2 shRNA plasmid. Next, to elucidate the mechanisms by which blockage of UCP2 ameliorated HG-induced EMT, we examined the role of UCP2 on the MMP in HK-2 cells by the confocal microscope. As shown in Figure 6(c), HG or UCP2 overexpression plasmid significantly induced decreased MMP. HG-induced MMP loss was rescued by cotreatment with UCP2 shRNA plasmid. We further explored the role of UCP2 on the mitochondrial morphology using Mitotracker. As shown in Figure 6(d), HG or UCP2 overexpression plasmid induced mitochondrial fragmentation, indicating the increased mitochondrial fission and dysfunction. In contrast, UCP2 shRNA plasmid treatment attenuated this trend induced by HG. Collectively, inhibition of c-Src/PPAR*γ*/UCP2 pathway significantly inhibited HG-induced EMT and mitochondrial dysfunction in HK-2 cells.

## 4. Discussion

DN is the leading cause of end-stage renal disease and contributes to increased mortality and economic burden worldwide. Multiple factors, such as metabolic abnormalities, inflammation, immunoregulation, and genetic predisposition, are involved in the process of DN, but the exact mechanism is not quite clarified [24]. It is now widely accepted that renal fibrosis is the most vital process for a variety of chronic kidney diseases that eventually turn into end-stage renal disease, including DN. Renal fibrosis, characterized by the abnormal accumulation of ECM, can manifest as glomerulosclerosis in the glomerulus and tubulointerstitial fibrosis mainly in the tubules [25, 26]. c-Src, a member of the Src kinase family, is activated by a number of stresses and implicated in the regulation of cellular processes in multiple tissues. c-Src can be activated in the kidneys of diabetic animals, glomerular mesangial cells, podocytes, and tubular epithelial cells exposed to HG [14, 15, 27]. Indeed, accumulating evidence suggested a strong correlation between c-Src and DN pathogenesis. Our previous study has demonstrated that c-Src activity was increased in kidneys of db/db mice and involved in renal tubular epithelial cells apoptosis in response to hyperglycemia in DN [15]. However, the role and molecular mechanisms of c-Src on renal fibrosis are yet to be elucidated. In this study, we explored the effect of c-Src inhibition on glomerulosclerosis and tubulointerstitial fibrosis in vivo and in vitro, respectively. In vivo, c-Src inhibition improved diabetes-induced glomerular hypertrophy, partial glomerulosclerosis, mesangial cells hyperplasia and hypertrophy, inflammatory cell infiltration, mesangial expansion, collagen deposition, tubular ectasia and cell swelling, and interstitial fibrous tissue proliferation in the diabetic kidneys. Similarly, c-Src inhibition could significantly alleviate renal fibrosis through decreasing protein levels of fibronectin, collagen IV, and *α*-SMA, as well as increasing protein level of E-cadherin. In vitro, HG-induced ECM accumulation, inflammation, and proliferation were inhibited in PP2-treated MMCs. Similarly, HG-induced EMT was also inhibited in PP2-treated HK-2 cells. These findings suggest that inhibition of c-Src can ameliorate diabetes-induced renal fibrosis.

Bioinformatic analysis is an efficient and reliable tool to identify potential therapeutic targets and diagnostic biomarkers for precision medicine. With the widespread application of high-throughput sequencing technology and public databases, bioinformatic analysis has been gradually used to study the molecular mechanisms of kidney diseases, such as membrane nephropathy, lupus, IgA nephropathy, and DN [28]. In this study, we performed bioinformatic analysis to screen the key genes and pathways related to DN and elucidated their molecular mechanism based on two GEO datasets. The GSE33744 dataset was used to identify the DEGs between nondiabetic and diabetic mice renal glomerular. A total of 839 DEGs were identified from GSE33744, including 579 upregulated genes and 260 downregulated genes. GO functional enrichment analysis showed that the upregulated genes were mainly enriched in signal transduction, membrane, and hydrolase activity, and the downregulated genes were mainly enriched in oxidation-reduction process, membrane, and metal ion binding. KEGG pathway enrichment analysis showed that the DEGs were mainly enriched in pathways in cancer, PI3K-Akt signaling pathway, neuroactive ligand-receptor interaction, MAPK signaling pathway, and Rap1 signaling pathway. Following PPI network analysis identified 23 functional modules and top 50 hub genes. In these 50 hub genes, the COX-2 pathway was selected for further investigation based on GO and KEGG enrichment analyses. Furthermore, the GSE86300 dataset was used to identify the DEGs between nondiabetic and diabetic mice renal cortex. A total of 238 DEGs were identified from GSE86300, including 112 upregulated genes and 126 downregulated genes. GO functional enrichment analysis showed that the upregulated genes were mainly enriched in signal transduction, extracellular exosome, and oxidoreductase activity, and the downregulated genes were mainly enriched in lipid metabolic process, membrane, and ATP binding. KEGG pathway enrichment analysis showed that the DEGs were mainly enriched in pathways in metabolic pathways, complement and coagulation cascades, Bile secretion, and PPAR signaling pathway. Following PPI network analysis identified 7 functional modules and top 50 hub genes. In these 50 hub genes, the UCP2 pathway was selected for further investigation based on GO and KEGG pathway enrichment analyses.

COX-2 is an inducible enzyme that catalyzes the conversion of arachidonic acid into prostaglandins which in turn cause pain, fever, and inflammation responses. Its expression can be induced by a number of inflammatory cytokines, mitogenic factors, and physical stimuli [29]. Previously, numerous studies revealed that COX-2 was increased in podocyte, glomerular mesangial cells, and renal tubular cells under HG condition, which contributed to the pathogenesis of DN [30–33]. Interestingly, the promoter region of the COX-2 gene contains several putative binding motifs for the binding of NF-*κ*B [34]. NF-*κ*B is a family of inducible transcription factors that mediates signal-induced expression of numerous genes involved in different biological processes, including immune response, inflammation, cellular transformation, cell proliferation, angiogenesis, invasion, and metastasis [35]. The activated and phosphorylated form of NF-*κ*B subunits (p65 and p50) translocates to the nucleus and thereby induces the expression of target genes related to DN [36]. However, almost nothing is known about the role of NF-*κ*B in modulating the COX-2 expression in DN. In this study, we found that the phosphorylation of NF-*κ*B subunit p65 and its downstream target gene COX-2 was significantly elevated in diabetic kidneys. These changes were reversed by treatment of c-Src inhibitor PP2. Meanwhile, c-Src inhibitor PP2 also inhibited HG-induced the phosphorylation of NF-*κ*B p65 and COX-2 expression in MMCs. In addition, NF-*κ*B inhibitor PDTC not only reduced HG-induced COX-2 expression but also dramatically abrogated HG-induced phosphorylation of c-Src. These results reveal c-Src or NF-*κ*B could act as an upstream regulator of COX-2 and a complex crosstalk between NF-*κ*B and c-Src. We then further examined the role of the c-Src/NF-*κ*B/COX-2 pathway on HG-induced ECM accumulation in MMCs. Our data demonstrated that pharmacological inhibition of c-Src with PP2, NF-*κ*B with PDTC, or COX-2 with NS-398 also resulted in the decreased expression of fibronectin and collagen IV. On the mechanism, prostaglandin E2 (PGE2) was reported to be involved in numerous physiological and pathological processes of the kidney via its four receptors [37]. PGE2 receptor (EP1 or EP2) deficiency markedly suppressed the extracellular matrix accumulation and COX-2 expression in mouse mesangial cells induced by TGF-*β*1 which is a pivotal mediator of renal fibrosis and glomerulosclerosis in diabetic nephropathy [38, 39]. Importantly, inhibition of COX-2 via a specific inhibitor celecoxib markedly blocked PGE2 production [40]. These findings suggested not only the important role of COX-2 in the expressions of extracellular matrix proteins in mesangial cells but also a complex crosstalk among COX-2, PGE2 and PGE2 receptors. Collectively, these results suggest that inhibition of the c-Src pathway ameliorated renal ECM accumulation and fibrosis at least in part through modulating the activity of the NF-*κ*B and expression of COX-2.

UCP2 is a mitochondrial inner membrane protein that uncouples the oxidative phosphorylation from ATP synthesis by dissipating the proton gradient generated across the mitochondrial inner membrane, therefore regulating glucose and lipid metabolism, immune cell activation, and ROS formation by mitochondria [41]. Consistently, previous studies have demonstrated that downregulation of UCP2 exacerbated oxidative stress damage as well as diabetes or HG-induced renal tubular epithelial cells apoptosis [42, 43]. However, a conflicting report suggested that UCP2 may act as a double-edged sword in renal tubular epithelial cell injury. Increased mitochondrial UCP-2 expression in renal proximal tubular cells increased O_2_ consumption and reduced O2 availability which subsequently contributed to diabetes-induced progressive kidney damage [44]. In this study, we found that the expression of UCP2 and its upstream gene PPAR*γ* was significantly elevated in diabetic kidneys. These changes were reversed by treatment with c-Src inhibitor PP2. Meanwhile, PP2 also inhibited HG-induced UCP2 and PPAR*γ* expression in HK-2 cells. In addition, PPAR*γ* antagonist GW9662 also reduced the HG-induced UCP2 expression. We further examined the role of the c-Src/PPAR*γ*/UCP2 pathway on HG-induced EMT in HK-2 cells. Our data demonstrated that HG induced EMT in HK-2 cells by increasing the mesenchymal marker and decreasing epithelial marker expression. The effect of HG on EMT was abolished by UCP2 shRNA plasmid. Additionally, UCP2 shRNA plasmid also prevented MMP loss and mitochondrial fragmentation induced by HG.

## 5. Conclusions

**Figure 7:**
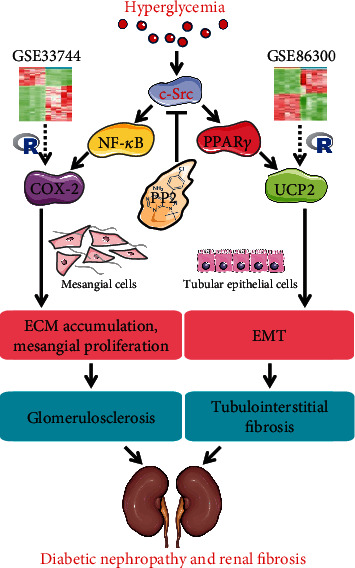
Schematic illustration of the mechanisms responsible for the PP2 effects on renal fibrosis in diabetic mice. PP2 decreased the expression of COX-2, phosphorylated p65, and fibrotic proteins, accompanied with attenuated renal glomerulosclerosis. PP2 also decreased the expression of PPAR*γ* and UCP2, accompanied with attenuated renal tubulointerstitial fibrosis and EMT.

In conclusion, we identified COX-2 as an important hub gene in renal glomerular between nondiabetic and diabetic mice through bioinformatic analysis. Administration of PP2 attenuated renal ECM accumulation and fibrosis via the NF-*κ*B/COX-2 pathway in the kidneys of diabetic mice. Moreover, PP2 ameliorates HG-induced ECM accumulation in MMCs by inhibiting NF-*κ*B activation and COX-2 expression in vitro. Furthermore, we identified UCP2 as an important hub gene in renal cortex between nondiabetic and diabetic mice through bioinformatic analysis. Administration of PP2 attenuated renal EMT and tubulointerstitial fibrosis via the PPAR*γ*/UCP2 pathway in the kidneys of diabetic mice. Moreover, PP2 ameliorated HG-induced EMT in HK-2 cells by inhibiting PPAR*γ* and UCP2 expression in vitro (Figure 7). Thus, c-Src inhibition may provide a potential therapeutic target for the treatment of DN.

## Figures and Tables

**Table 1:** The detailed information on the GO analysis of the upregulated and downregulated DEGs in GSE33744.

Category	Term	Count	*p* value
Upregulated			
BP	Signal transduction	45	0.007753334
BP	Multicellular organism development	37	0.016000738
BP	Cell adhesion	28	4.97064*E*-05
BP	Ion transport	27	0.002019604
BP	Proteolysis	25	0.007536866
BP	Positive regulation of cell proliferation	23	0.012198374
BP	Positive regulation of apoptotic process	22	6.66135*E*-05
BP	Inflammatory response	22	9.69908*E*-05
BP	Immune system process	21	0.001040971
BP	Intracellular signal transduction	18	0.017213392
CC	Membrane	226	1.18309*E*-07
CC	Integral component of membrane	187	0.038634939
CC	GO: 0005737 ~ cytoplasm	180	0.045971674
CC	Plasma membrane	148	0.001626062
CC	Extracellular region	95	1.44303*E*-13
CC	Extracellular exosome	90	0.001132912
CC	Extracellular space	65	7.97336*E*-06
CC	Integral component of plasma membrane	46	0.000743085
CC	Cell surface	29	0.001618609
CC	Cell junction	29	0.009876677
MF	Hydrolase activity	49	0.042624904
MF	Calcium ion binding	29	0.00687708
MF	Actin binding	24	9.88949*E*-06
MF	Peptidase activity	22	0.015404845
MF	Protein heterodimerization activity	21	0.027455776
MF	Metallopeptidase activity	13	0.000561141
MF	Heparin binding	12	0.001196627
MF	Metalloendopeptidase activity	11	0.000858787
MF	Calmodulin binding	10	0.034548521
MF	Hormone activity	8	0.0197492
Downregulated			
BP	Oxidation-reduction process	19	0.000443777
BP	Lipid metabolic process	17	4.48872*E*-05
BP	Response to drug	10	0.012221837
BP	Fatty acid metabolic process	8	0.001618978
BP	Hippocampus development	6	0.001202789
BP	Steroid metabolic process	6	0.002245487
BP	Fatty acid biosynthetic process	5	0.008687078
BP	Cholesterol metabolic process	5	0.016263715
BP	Protein glycosylation	5	0.038138181
BP	Phospholipid metabolic process	4	0.010702838
CC	Membrane	99	0.000388674
CC	Cytosol	37	0.000119979
CC	Perinuclear region of cytoplasm	18	0.001140012
CC	Cell junction	17	0.004153908
CC	Neuronal cell body	15	0.001706257
CC	Synapse	12	0.018930533
CC	Postsynaptic membrane	8	0.009523669
CC	Postsynaptic density	8	0.013861599
CC	Growth cone	6	0.027381838
CC	Terminal Bouton	5	0.032102973
MF	Metal ion binding	48	0.045332685
MF	ATP binding	26	0.026765145
MF	Transferase activity	25	0.035427208
MF	Oxidoreductase activity	16	0.00299816
MF	Transferase activity, transferring glycosyl groups	9	0.002247304
MF	Symporter activity	5	0.035838809
MF	Transferase activity, transferring hexosyl groups	4	0.004770367
MF	Extracellular ligand-gated ion channel activity	4	0.010436623
MF	Sodium-independent organic anion transmembrane transporter activity	3	0.03565372
MF	Glucuronosyltransferase activity	3	0.040654245

BP: biological process; CC: cellular component; MF: molecular function.

**Table 2:** The detailed information on the KEGG enrichment analysis of DEGs in GSE33744.

Term	Pathway	Count	*p* value	Genes
mmu05200	Pathways in cancer	27	0.003017125	ITGB1, CDKN1A, LAMA1, LPAR1, ADCY1, LPAR4, RASGRP2, PTGS2, EGFR, GLI3, ADCY5, FGF6, GNG2, BID, RUNX1T1, FZD3, FZD2, PTCH1, MMP2, PTCH2, VEGFB, BMP2, TRAF4, CCNE1, COL4A6, GNB4, CYCS
mmu04151	PI3K-Akt signaling pathway	23	0.010735996	ITGB1, CDKN1A, ANGPT2, ANGPT1, LAMA1, NOS3, FLT4, TNC, VEGFB, LPAR1, IL4RA, LPAR4, EPOR, EGFR, FGF6, NR4A1, GNG2, CCNE1, GNB4, COL4A6, ITGB6, TLR4, ITGA9
mmu04080	Neuroactive ligand-receptor interaction	20	0.009368143	GABRB3, GABRB1, CHRNA4, GRID1, CHRNA7, P2RY14, GABRA4, C5AR1, LPAR1, LPAR4, NR3C1, ADRA1A, P2RX7, GLRA2, CALCR, CNR1, HRH2, P2RX1, ADORA3, TSHB
mmu04010	MAPK signaling pathway	17	0.024073268	NTRK2, MEF2C, GADD45B, PLA2G4B, CACNA2D4, RASGRP2, EGFR, CACNA1G, FGF6, NR4A1, CACNG7, CACNB3, MAP3K8, MAP3K6, HSPA1B, MAP4K4, HSPA1A
mmu04015	Rap1 signaling pathway	15	0.027634917	ITGB1, ANGPT2, ANGPT1, FLT4, VEGFB, LPAR1, ADCY1, LPAR4, RASGRP2, EGFR, ADCY5, FGF6, CNR1, PRKD3, RAPGEF5
mmu04014	Ras signaling pathway	15	0.044994767	PLA2G12B, ANGPT2, PLA2G2E, ANGPT1, PLA2G4B, FLT4, VEGFB, GAB2, RASGRP2, EGFR, FGF6, GNG2, GNB4, RIN1, RAPGEF5
mmu04921	Oxytocin signaling pathway	13	0.009629191	RYR1, MEF2C, CDKN1A, NOS3, PLA2G4B, NFATC2, CACNA2D4, ADCY1, COX-2, EGFR, ADCY5, CACNG7, CACNB3
mmu00230	Purine metabolism	13	0.033776013	ENTPD1, ENTPD2, PDE6H, GDA, PDE1A, AK1, NME4, NME5, ADCY1, ADCY5, ADSSL1, ENPP3, PDE8B
mmu04020	Calcium signaling pathway	13	0.03503903	RYR1, NOS3, PDE1A, CHRNA7, ADCY1, ADRA1A, EGFR, CACNA1G, P2RX7, SLC8A3, GNA14, HRH2, P2RX1
mmu00590	Arachidonic acid metabolism	12	0.000415	PTGIS, PLA2G2E, PLA2G12B, CYP2J13, PLA2G4B, GPX7, ALOX12B, CYP4A14, LTC4S, COX-2, PTGDS, PTGS1
mmu05414	Dilated cardiomyopathy	11	0.000926	ITGB1, CACNG7, CACNB3, TNNT2, TPM1, DAG1, CACNA2D4, ITGB6, ADCY1, ADCY5, ITGA9
mmu04726	Serotonergic synapse	11	0.024426847	GABRB3, GABRB1, GNG2, CYP2J13, KCND2, PLA2G4B, GNB4, ALOX12B, COX-2, ADCY5, PTGS1
mmu04976	Bile secretion	9	0.004518526	ABCC3, SLCO1A1, SLCO1A5, ADCY1, SLC51A, ABCB1A, SLC22A7, ABCB1B, ADCY5
mmu05410	Hypertrophic cardiomyopathy	9	0.008596921	ITGB1, CACNG7, CACNB3, TNNT2, TPM1, DAG1, CACNA2D4, ITGB6, ITGA9
mmu04512	ECM-receptor interaction	9	0.015941382	ITGB1, SV2B, LAMA1, DAG1, TNC, COL4A6, GP1BA, ITGB6, ITGA9
mmu05032	Morphine addiction	9	0.021582369	GABRB3, GABRB1, GNG2, GABRA4, PDE1A, GNB4, ADCY1, PDE8B, ADCY5
mmu04066	HIF-1 signaling pathway	9	0.035057542	CDKN1A, ANGPT2, PFKFB3, ANGPT1, NOS3, TRF, TLR4, EGFR, PDK1
mmu04723	Retrograde endocannabinoid signaling	9	0.036841276	GABRB3, GABRB1, GNG2, CNR1, GABRA4, GNB4, ADCY1, COX-2, ADCY5
mmu05412	Arrhythmogenic right ventricular cardiomyopathy	8	0.011585663	ITGB1, CACNG7, CACNB3, DAG1, DSG2, CACNA2D4, ITGB6, ITGA9
mmu04727	GABAergic synapse	8	0.04190812	GABRB3, GABRB1, GNG2, GABRA4, GNB4, ADCY1, HAP1, ADCY5
mmu00982	Drug metabolism-cytochrome P450	7	0.034801983	ALDH1A3, ADH1, AOX4, GSTO1, UGT2B37, AOX1, UGT2A3
mmu00532	Glycosaminoglycan biosynthesis	6	0.000661	CHST7, CHST11, CHST12, CHPF2, CHST15, CHSY3
mmu05217	Basal cell carcinoma	6	0.048717123	BMP2, FZD3, FZD2, PTCH1, PTCH2, GLI3

**Table 3:** The degree values of the top 50 hub genes in GSE33744.

Gene	Gene title	Score	LogFC
EGFR	Epidermal growth factor receptor	52	-1.4
COX-2	Cyclooxygenase-2	34	2.019
TLR4	Toll-like receptor 4	31	1.361
GNG2	Guanine nucleotide binding protein (G protein) gamma 2	30	1.84
GNB4	Guanine nucleotide binding protein (G protein) beta 4	28	1.235
ADCY5	Adenylate cyclase 5	22	1.038
ADCY1	Adenylate cyclase 1	21	1.206
CCL6	Chemokine (C-C motif) ligand 6	21	2.879
ITGB1	Integrin beta 1	21	1.11
NTRK2	Neurotrophic tyrosine kinase, receptor, type 2	19	1.575
IL18	Interleukin 18	19	1.324
QSOX1	Quiescin Q6 sulfhydryl oxidase 1	19	1.103
HSPA1B	Heat shock protein 1B	18	-3.42
ENTPD1	Ectonucleoside triphosphate diphosphohydrolase 1	18	1.853
MMP2	Matrix metallopeptidase 2	18	1.698
PF4	Platelet factor 4	18	2.06
SLCO1A1	Solute carrier organic anion transporter family, member 1a1	16	-4.82
UGT2B37	UDP glucuronosyltransferase 2 family, polypeptide B37	16	-1.48
ANGPT1	Angiopoietin 1	16	-1.12
LPAR1	Lysophosphatidic acid receptor 1	16	1.231
KALRN	Kalirin, RhoGEF kinase	16	1.014
CYP4A14	Cytochrome P450, family 4, subfamily a, polypeptide 14	16	3.522
ISL1	ISL LIM Homeobox 1	16	1.93
NOS3	Nitric oxide synthase 3	16	1.159
C5AR1	Complement component 5a receptor 1	16	3.044
CP	Veruloplasmin	16	1.134
TRF	Transferrin	15	1.563
PCSK9	Proprotein convertase subtilisin/kexin type 9	15	-1.68
NES	Nestin	15	1.083
EXO1	Exonuclease 1	15	1.913
FBN1	Fibrillin 1	15	1.168
UGT2A3	UDP glucuronosyltransferase 2 family, polypeptide A3	14	-1.95
GPR143	G protein-coupled receptor 143	14	1.294
PTGS1	Prostaglandin-endoperoxide synthase 1	14	1.212
P2RY14	Purinergic receptor P2Y, G-protein coupled, 14	14	1.788
ENPP3	Ectonucleotide pyrophosphatase/phosphodiesterase 3	13	-1.31
CNR1	Cannabinoid receptor 1 (brain)	13	-1.58
GNA14	Guanine nucleotide binding protein, alpha 14	13	-1.38
P2RX1	Purinergic receptor P2X 1	13	1.223
EDN2	Endothelin 2	13	1.312
GRP	Gastrin releasing peptide	13	2.796
CYP2J13	Cytochrome P450, family 2, subfamily j, polypeptide 13	13	-2.43
TNNT3	Troponin T3, fast skeletal type	13	3.247
HP	Haptoglobin	13	3.071
BMP2	Bone morphogenetic protein 2	13	1.283
SNAI2	Snail family zinc finger 2	13	1.057
NEGR1	Neuronal growth regulator 1	13	-1.84
FSTL1	Follistatin-like 1	13	1.575
ADM	Adrenomedullin	12	1.308
ABCB1A	ATP-binding cassette, subfamily B (MDR/TAP), member 1A	12	2.014

**Table 4:** The detailed information on the GO analysis of the upregulated and downregulated DEGs in GSE86300.

Category	Term	Count	*p* value
Upregulated			
BP	Signal transduction	13	0.029093438
BP	Oxidation-reduction process	9	0.024404362
BP	Lipid metabolic process	8	0.00998742
BP	Negative regulation of apoptotic process	8	0.028039906
BP	Intracellular signal transduction	6	0.057525068
BP	DNA replication	5	0.003880893
BP	Positive regulation of ERK1 and ERK2 cascade	5	0.01671187
BP	Small GTPase mediated signal transduction	5	0.034683472
BP	Negative regulation of lipoprotein lipase activity	4	2.69*E*-06
BP	Triglyceride homeostasis	4	3.64*E*-04
CC	Extracellular exosome	25	0.00346128
CC	Extracellular region	23	5.66*E*-05
CC	Extracellular space	18	0.00145449
CC	Cytosol	16	0.035864717
CC	Intracellular membrane-bounded organelle	11	0.004880998
CC	Cell surface	9	0.014877768
CC	Blood microparticle	6	5.91*E*-04
CC	Apical plasma membrane	6	0.026095659
CC	Basolateral plasma membrane	4	0.084936357
CC	Extracellular vesicle	3	0.025790832
MF	Oxidoreductase activity	8	0.048891279
MF	Calcium ion binding	8	0.090469254
MF	Enzyme inhibitor activity	4	0.001278238
MF	Phospholipid binding	4	0.012646826
MF	Heparin binding	4	0.05071775
MF	Heme binding	4	0.072179828
MF	ATPase activity	4	0.098135899
MF	Fatty acid binding	3	0.008936699
MF	Glutathione transferase activity	3	0.014161492
MF	ATPase activity, coupled to transmembrane movement of substances	3	0.029804054
Downregulated			
BP	Lipid metabolic process	16	9.41*E*-08
BP	Oxidation-reduction process	15	0.0000495
BP	Cell differentiation	9	0.094964371
BP	Spermatogenesis	6	0.096883326
BP	Steroid metabolic process	5	0.001609187
BP	Fatty acid metabolic process	5	0.014290964
BP	Regulation of blood coagulation	4	0.000187
BP	Hippocampus development	4	0.009520707
BP	Protein folding	4	0.041356989
BP	Organic anion transport	3	0.003069172
CC	Membrane	62	3.31*E*-05
CC	Extracellular region	22	8.29*E*-04
CC	Endoplasmic reticulum	14	0.037877677
CC	Extracellular space	14	0.085953795
CC	Intracellular membrane-bounded organelle	13	0.001184947
CC	Perinuclear region of cytoplasm	9	0.044162931
CC	Endoplasmic reticulum membrane	9	0.04998487
CC	Cell surface	8	0.068104588
CC	Growth cone	5	0.013026472
CC	Organelle membrane	4	0.016577263
MF	ATP binding	16	0.038783118
MF	Oxidoreductase activity	14	7.50*E*-05
MF	Calcium ion binding	9	0.06266387
MF	Heme binding	5	0.022041571
MF	Organic anion transmembrane transporter activity	4	3.09*E*-04
MF	Calcium-dependent protein binding	4	0.00854366
MF	Unfolded protein binding	4	0.010726793
MF	Metallopeptidase activity	4	0.073836747
MF	Sodium-independent organic anion transmembrane transporter activity	3	0.011626112
MF	Heat shock protein binding	3	0.041228079

BP: biological process; CC: cellular component; MF: molecular function.

**Table 5:** The detailed information on the KEGG enrichment analysis of DEGs in GSE86300.

Term	Pathway	Count	P value	Genes
mmu01100	Metabolic pathways	24	0.013965513	RRM2, MAOB, ACSM3, UGT8A, ST8SIA1, ALOX15, CYP4A14, LTC4S, CYP51, HSD11B1, CYP24A1, BDH1, ALDH1B1, CYP2J13, MTHFD1L, KYNU, HSD17B2, AGPS, UGT2A3, PLCH1, HMGCS2, DEGS2, IDO2, ACOT3
mmu04610	Complement and coagulation cascades	8	2.33E-05	C3, CPB2, FGG, MASP2, MASP1, C8A, CD55, MBL2
mmu04976	Bile secretion	6	0.001258086	ABCC4, SLCO1A4, SLCO1A1, EPHX1, ABCB1A, SLC22A7
mmu03320	PPAR signaling pathway	6	0.002143511	FABP1, LPL, CYP4A14, ANGPTL4, CD36, RXRG
mmu05150	S. aureus infection	5	0.002467676	C3, FGG, MASP2, MASP1, MBL2
mmu05134	Legionellosis	5	0.003983622	PYCARD, C3, NAIP1, HSPA1B, HSPA1A
mmu00980	Metabolism of xenobiotics by cytochrome P450	5	0.006031624	HSD11B1, GSTO1, EPHX1, UGT2A3, GSTM6
mmu00982	Drug metabolism-cytochrome P450	5	0.006724748	MAOB, GSTO1, UGT2A3, FMO5, GSTM6
mmu00140	Steroid hormone biosynthesis	5	0.017339009	HSD11B1, SRD5A2, HSD17B2, UGT2A3, CYP7B1
mmu05204	Chemical carcinogenesis	5	0.02085394	HSD11B1, GSTO1, EPHX1, UGT2A3, GSTM6
mmu00650	Butanoate metabolism	4	0.003471535	BDH1, ACSM3, HMGCS2, AACS
mmu00380	Tryptophan metabolism	4	0.016335965	MAOB, ALDH1B1, KYNU, IDO2

**Table 6:** The degree values of the top 50 hub genes in GSE86300.

Gene	Gene title	Score	LogFC
Fgg	Fibrinogen gamma chain	3781	1.945439
C8a	Complement component 8, alpha polypeptide	3614	-2.32194
Gc	Group specific component	3517	2.661948
Hrg	Histidine-rich glycoprotein	3437	-2.73542
Tm4sf4	Transmembrane 4 superfamily member 4	2904	-1.7815
Mbl2	Mannose-binding lectin (protein C) 2	1824	1.632683
Cpb2	Carboxypeptidase B2 (plasma)	1815	1.143985
Fabp1	Fatty acid binding protein 1, liver	1498	2.670807
Angptl3	Angiopoietin-like 3	1489	1.208791
C3	Complement component 3	562	1.982437
Pzp	Pregnancy zone protein	290	-2.58665
Masp1	Mannan-binding lectin serine peptidase 1	246	2.101058
Masp2	Mannan-binding lectin serine peptidase 2	246	-1.38516
Dscc1	DNA replication and sister chromatid cohesion 1	162	1.272575
Mcm5	Minichromosome maintenance complex component 5	162	1.07099
Apoe	Apolipoprotein E	158	1.551564
Ncapg2	Non-SMC condensin II complex, subunit G2	152	1.352226
Rrm2	Ribonucleotide reductase M2	150	1.020213
Trf	Transferrin	144	1.059667
Kif20b	Kinesin family member 20B	132	-1.50486
Arhgap11a	Rho GTPase activating protein 11A	129	1.365888
Apoc1	Apolipoprotein C-I	54	1.28042
Ugt2a3	UDP glucuronosyltransferase 2 family, polypeptide A3	39	-1.37089
Hsph1	Heat shock 105 kDa/110 kDa protein 1	38	-1.06227
Pcsk9	Proprotein convertase subtilisin/kexin type 9	38	-1.11691
Hspa1a	Heat shock protein 1A	36	-2.39622
Hspa1b	Heat shock protein 1B	34	-1.87672
Chaf1b	Chromatin assembly factor 1 subunit B (p60)	34	1.093252
Dnaja4	DnaJ heat shock protein family (Hsp40) member A4	33	-1.03221
Lpl	Lipoprotein lipase	32	-1.02081
Dnajb6	DnaJ heat shock protein family (Hsp40) member B6	24	-1.08687
Cyp4a14	Cytochrome P450, family 4, subfamily a polypeptide 14	16	2.444874
Cd36	CD36 antigen	15	-2.11604
Kynu	Kynureninase (L-kynurenine hydrolase)	13	1.90505
Hmgcs2	3-hydroxy-3-methylglutaryl-coenzyme A synthase 2	12	3.639891
Blm	Bloom syndrome, RecQ helicase-like	12	1.117173
Slco1a1	Solute carrier organic anion transporter family member 1a1	10	-4.71252
Angptl4	Angiopoietin-like 4	10	1.562673
Slc22a7	Solute carrier family 22 (organic anion transporter) member 7	9	-5.16673
Ucp2	Uncoupling protein 2 (mitochondrial, proton carrier)	9	1.274927
Fmo5	Flavin containing monooxygenase 5	9	-2.31674
Slco1a4	Solute carrier organic anion transporter family member 1a4	7	-1.87469
Cyp2j13	Cytochrome P450 family 2 subfamily j polypeptide 13	7	-1.10038
Angptl8	Angiopoietin-like 8	7	1.356146
Ido2	Indoleamine 2,3-dioxygenase 2	7	-2.04659
Hist2h3c2	Histone cluster 2 H3c2	7	-3.04114
Abcb1a	ATP-binding cassette, subfamily B (MDR/TAP) member 1A	6	1.424367
Ahsa2	AHA1, activator of heat shock protein ATPase 2	6	-1.45842
Maob	Monoamine oxidase B	6	1.392949
Gas2l3	Growth arrest-specific 2 like 3	6	1.321103

## Data Availability

This study benefited from the Cancer Genome Atlas (TCGA) and GEO databases. We appreciate the data platform, and the authors uploaded their data.
